# Improved cooling of photovoltaic panels by natural convection flow in a channel with adiabatic extensions

**DOI:** 10.1371/journal.pone.0302326

**Published:** 2024-07-11

**Authors:** Nacer Badi, Ali Hatem Laatar

**Affiliations:** 1 Thermal Management and Sustainable Research Laboratory, Department of Physics, Faculty of Science, University of Tabuk, Tabuk, Saudi Arabia; 2 Renewable Energy and Environmental Technologies Research Center, University of Tabuk, Tabuk, Saudi Arabia; 3 Laboratory of Energetics and Thermal and Mass Transfer (LR01ES07), Faculty of Sciences of Tunis, University of Tunis El Manar, Tunis, Tunisia; 4 Department of Physics, Faculty of Sciences of Bizerte, University of Carthage, Jarzouna, Tunisia; GH Raisoni College of Engineering and Management Pune, INDIA

## Abstract

In hot dry regions, photovoltaic modules are exposed to excessive temperatures, which leads to a drop in performance and the risk of overheating. The present numerical study aims to evaluate the natural air cooling of PV modules by an inclined chimney mounted at the back. The basic equations were solved using the finite volume method. The validity of the model is verified by comparison with the data available in the literature. Thermal and dynamic flow patterns are analyzed for a variety of parameters: Rayleigh numbers from 10^2^ to 10^6^, PV panel tilt angle from 15° to 90°, and channel aspect ratios from 1/20 to 1/5. A critical aspect ratio has been determined to minimize overheating of the PV module. According to the computational results, the tilt angle and modified Rayleigh number increase the mass flow rate and mean Nusselt number. The overheating zone with maximum temperatures is located in the upper part of the photovoltaic panel. The addition of an extension to both channel’s inlet and outlet was found to improve the cooling of the photovoltaic panels; however, only the extensions downstream of the channel are truly effective. The critical lengths at which channel performance improves significantly were identified by examining the impact of longer extensions on channel performance. Increasing the extension length from 0 to 3H improves the mass flow rate by 65%, the average Nusselt number by 13.4%, and leads to an 11% decrease in maximum temperature when Ra* = 10^6^. This cooling technique is particularly promising for hot dry regions where water is scarce.

## 1. Introduction

Solar energy is receiving considerable attention as a sustainable and renewable energy source that can mitigate climate change by reducing greenhouse gas emissions. This energy source is particularly attractive in hot, desert regions with high solar radiation, as it can provide abundant electricity [1]. However, implementing solar energy in these regions presents significant challenges. The climate in these regions is characterized by hot, dry air, with temperatures exceeding 50°C during the day and dropping to 10–20°C at night. These extreme climatic conditions subject photovoltaic installations to high thermal stresses throughout the day, affecting their performance, reducing their life span, and even causing irreversible damage [2]. In addition, dust and sand in the air can accumulate on photovoltaic (PV) modules, reducing their efficiency. In addition, desert regions pose logistical problems for the solar systems’ installation and maintenance, given their geographic isolation and lack of infrastructure. It is therefore crucial to adapt the appropriate cooling technique to these extreme conditions in order to protect PV modules from degradation and improve their performance [3].

Several studies show that photovoltaic modules typically convert about 20% of solar energy into electricity, while the remaining 80% is converted into heat. This excess of heat causes the PV cells’ temperature to rise, leading to reduced electricity production and increased heat production, which can negatively impact conversion efficiency [4]. Single-junction solar cell efficiency may reach up to 25%, depending on the technology in place [5]. Installing PV modules in hot regions can exacerbate the temperature difference between the module and the ambient air, resulting in reduced electrical energy production and accelerated module degradation. For instance, the temperature difference between a PV module and the surrounding air can exceed 40°C under such conditions [6].

To prevent a drop in efficiency, it is crucial to keep the temperature of the photovoltaic cells as low as possible, which can be achieved by implementing various cooling solutions or thermal management systems. Cooling techniques are often classified into three main categories: active, passive, and hybrid methods. Each of these methods has its advantages and disadvantages [7].

Passive cooling techniques utilize natural processes such as air convection, water flow, or thermal conduction to dissipate heat without consuming additional energy. These techniques include phase change materials, liquid immersion, heat pipes, microchannels, impingement jets, microporous evaporation sheets, and cotton wicks. On the other hand, active cooling methods require additional equipment such as fans and pumps to remove heat and force the circulation of cooling fluids like air, water, or nanofluids [3]. Forced circulation methods can maintain acceptable PV cell temperatures by achieving a high mass flow rate, but the additional equipment and energy consumption can make this approach more complicated and expensive [7]. Water is the most common active cooling medium for PV panels, while air is the most abundant natural cooling medium for passive cooling. However, air is not typically used for cooling PV panels due to its low thermal conductivity and slow heat transfer rate. Hybrid cooling systems have become a leading trend in photovoltaic module cooling research [8]. These innovative approaches combine several thermal management techniques, including passive and active cooling methods. Examples of these hybrid cooling systems include air-water cooling, air-fine cooling, phase change material (PCM)-water cooling, PCM-air cooling, PCM-fine cooling, PCM + thermal dissipator, PCM-metal foam, thermal dissipator + thermal conduit, gas-liquid cooling, and air cooling with heat exchanger [9].

Despite being less effective than other cooling methods in dissipating heat, passive cooling is a simpler, lighter, and cheaper alternative. Moreover, passive cooling techniques can be enhanced with diffusers or heat sinks. As passive cooling technology improves in terms of thermal efficiency, it could become a more viable option for efficiently harnessing solar energy.

The literature contains a vast and diverse range of research works exploring various cooling methods for photovoltaic panels including active cooling, passive cooling, hybrid cooling, and passive air cooling [7–9]. In the following, we will focus on passive air cooling technology as it is directly related to the present work.

The most economical and easiest way to cool PV modules is to use natural convection airflow under the panels. This technique relies on the chimney effect to draw warm air through a channel behind the PV modules. However, the effectiveness of this cooling approach is limited by the low heat transfer rate of natural convection and the low thermal conductivity and volumetric heat capacity of air. Air-cooled photovoltaic/thermal (PV/T) systems as well as building-integrated PV systems (BIPV) are concrete examples of cooling PV modules by natural air convection with a suitable energy efficiency. They are integrated into sloping roofs or facades of buildings for urban solar applications. In BIPV applications, an air gap is used to circulate air to cool the PV modules and the preheated air can be used for the thermal needs of the building. Ventilated PV/T is a viable and economical method of providing electrical power, warm air, and hot water to buildings. The air gap in a BIPV system behaves like a natural air stream, driven by buoyancy and wind-induced pressure difference. Several studies have reported low induced air velocities, less than 0.5 m s^-1^, in the air channel behind the PV module justifying the fact that the flow is mainly laminar.

Brinkworth et al investigated a method of lowering the temperature of photovoltaic cells using natural convection airflow in a duct at the back of the panel [10]. Simulations determined fundamental parameters such as the height and depth of the cooling duct, which was found to be effective in reducing the temperature of the solar cells by 15 to 20 K. This technique has proven effective in improving the energy output of PV coatings used in building walls and roofs.

Tripanagnostopoulos and Themelis experimentally tested three passive systems for cooling photovoltaic panels by natural convection [11]. The first system includes an air duct at the rear of the PV panel; the second system contains an air duct similar to the first, in which a thin sheet metal (TMS) has been inserted to increase the cooling process; while the third system, it is formed by metal fins placed at the back of the PV panel to increase the heat exchange surface. The test results show that the last cooling system (fins) is the most efficient, followed by the second solution (TMS), then the first.

Baloch et al. conducted an experiment in which they compared the performance of two photovoltaic (PV) panels in a hot climate in Saudi Arabia [12]. One panel was placed in a normal frame, while the other was cooled by a duct channel. The experiment took place during the second half of the year. The results showed that in June and December, the temperature decreased from 71 °C to 48 °C and from 45 °C to 36 °C, respectively. The proposed cooling system resulted in an average increase of over 35% in power output and a relative reduction of 19% in levelized energy cost.

To prevent photovoltaic panels from overheating in hot climates, Abd-Elhady et al. have proposed a passive cooling solution using natural convection [13]. The method involves drilling holes in the photovoltaic panels to allow the hot air beneath the panels to escape. This air is replaced by cooler ambient air, ensuring better cooling of the PV panel. Experimental tests validated the concept, followed by simulations that explored the effects of hole size and quantity. A critical hole diameter was identified, beyond which smaller holes led to an increase in panel temperature.

Glick et al. conducted research that differs from most previous studies which centered on determining the optimal tilt angle for PV panels to maximize sunlight absorption [14]. Instead, their focus was on the impact of the PV panel arrangement on the incoming wind flow. By varying the tilt angle of the PV panels and calculating the heat transfer coefficients of their surfaces, the researchers found that a 30° tilt angle resulted in the highest heat transfer rate on the bottom surface. They therefore suggested improving the flow of sub-panels through height optimization or implementing baffles to enhance the lifespan and efficacy of the PV panels.

Naghavi et al. conducted experimental and numerical studies to investigate the impact of natural convection on the temperature reduction of PV panels [15]. They investigated the effect of the distance between the panels and the roof on the performance of the PV system. Their experiments showed that the average temperature of the PV module without an air gap was 12 ± 5°C higher than of modules with air gaps greater than 200 mm. The CFD simulation revealed that the surface temperatures of the panels decreased with increasing air gaps and the maximum efficiency of the PV panel was 15.44% for 250 mm spacing.

Zhou et al. examined a passive cooling method using vortex generators (VGs) on photovoltaic (PV) modules for free convection conditions [16]. The VGs were thermally non-conductive and placed in an array on the rear surface of the module. The study found that VGs reduced module operating temperatures up to 2°C under free convection and 3°C with conductive materials. Computational fluid dynamics and PIV measurements showed that VG-induced mixing in the boundary layer increased convective heat transfer. The study highlights that the VGs’ shape and placement are essential and suggests that this cooling method could increase energy yield and prolong module lifespan, particularly in hot climates.

Sutanto et al. investigated the development of floating photovoltaic (PV) systems with passive cooling using thermosyphon to increase their power output [17]. The results show that the power output of floating PV systems is about 4.52% higher than that of ground-mounted PV installations. The introduction of a thermosyphon cooling system increases the power output of floating PV panels by about 7.86% compared to ground-mounted PV generation and by 3.34% compared to floating PV systems. The results of the study highlight the potential of using floating PV systems with passive cooling to address the problem of limited land availability and minimize environmental impacts while meeting global energy demand.

Fatnassi et al. performed particle image velocimetry (PIV) experiments to predict the morphology of natural convection-induced fluid flow in an inclined double-skin photovoltaic (IPVDSF) facade during transient and laminar regimes [18]. The experiments were performed under realistic geometric and environmental conditions, and an irregularly heated channel was used with three different slopes (-10°, 0°, and +10°) for the PVDSF duct. The results showed that the slope has a significant effect on the flow and velocity fields for both flow regimes, and the study aimed to provide baseline data for numerical results and design development of such systems while considering aesthetics and PV efficiency improvement aspects.

Panda et al. conducted experiments to investigate the effect of front or rear cooling of a PV module on its performance [19]. They cooled the front of PV modules by circulating water at a specific mass rate while cooling the back with wet grass. The average reduction in panel temperature was 21.23°C, resulting in a 28.6% increase in power output and an average efficiency of 34.96%. Both cooling methods were effective, but top-side cooling was the best.

This literature review has shown that each PV module cooling technique has its characteristics in terms of energy efficiency, initial investment, and maintenance costs.

In hot, desert regions, water is scarce, and passive air-cooling techniques become very attractive, despite their low efficiency. These methods have the advantage of being economical, silent, easy to install, and maintenance-free.

The use of a duct at the back of the photovoltaic module is an old passive panel cooling technique. Several researchers have taken up this configuration (simple channel). El Kharaz et al. [20] used the CFD code ANSYS-Fluent to simulate turbulent flow in a vertical channel behind a photovoltaic panel. They used several turbulence models to investigate the impact of channel geometry on flow topology and heat exchange. They suggested optimal spacings between channel plates for heat fluxes from 10 to 300 W/m^2^. The findings revealed an average heat transfer coefficient Nu_a_ ranging from 70.9 to 132.8.

This study aims to investigate the use of natural convection for cooling photovoltaic panels. More specifically, we analyzed the concept of using an inclined chimney as a passive cooling system from the rear of photovoltaic panel. The chimney is a rectangular duct that passes through the back of the panel and extends to the channel outlet. Airflow in the channel is induced solely by buoyancy forces. This configuration has been designed on the basis of various studies of natural convection flows in parallel-walled heated channels. Most of these studies concern flows in vertical geometries, as in the case of electronic component cooling [21] or natural building ventilation [22]. However, there are a number of studies on natural convection in inclined channels with heated walls [23, 24], aimed at improving the performance of rooftop solar chimneys or roof-mounted solar panels. Other authors have studied mixed convection using fins to cool the channel [25].

In this work, we modified the simple channel to make it more efficient. Novelty design consists in placing adiabatic extensions upstream and downstream of the PV panel in order to increase the chimney draft by accelerating the airflow behind the module, thus improving the evacuation of the heat released by the PV panel. The aim of the study is to optimize the width of the channel and the length of the extensions as a function of the angle of inclination and the modified Rayleigh number, in order to improve the thermal performance of the system.

## 2. Problem formulation

**Fig 1:**
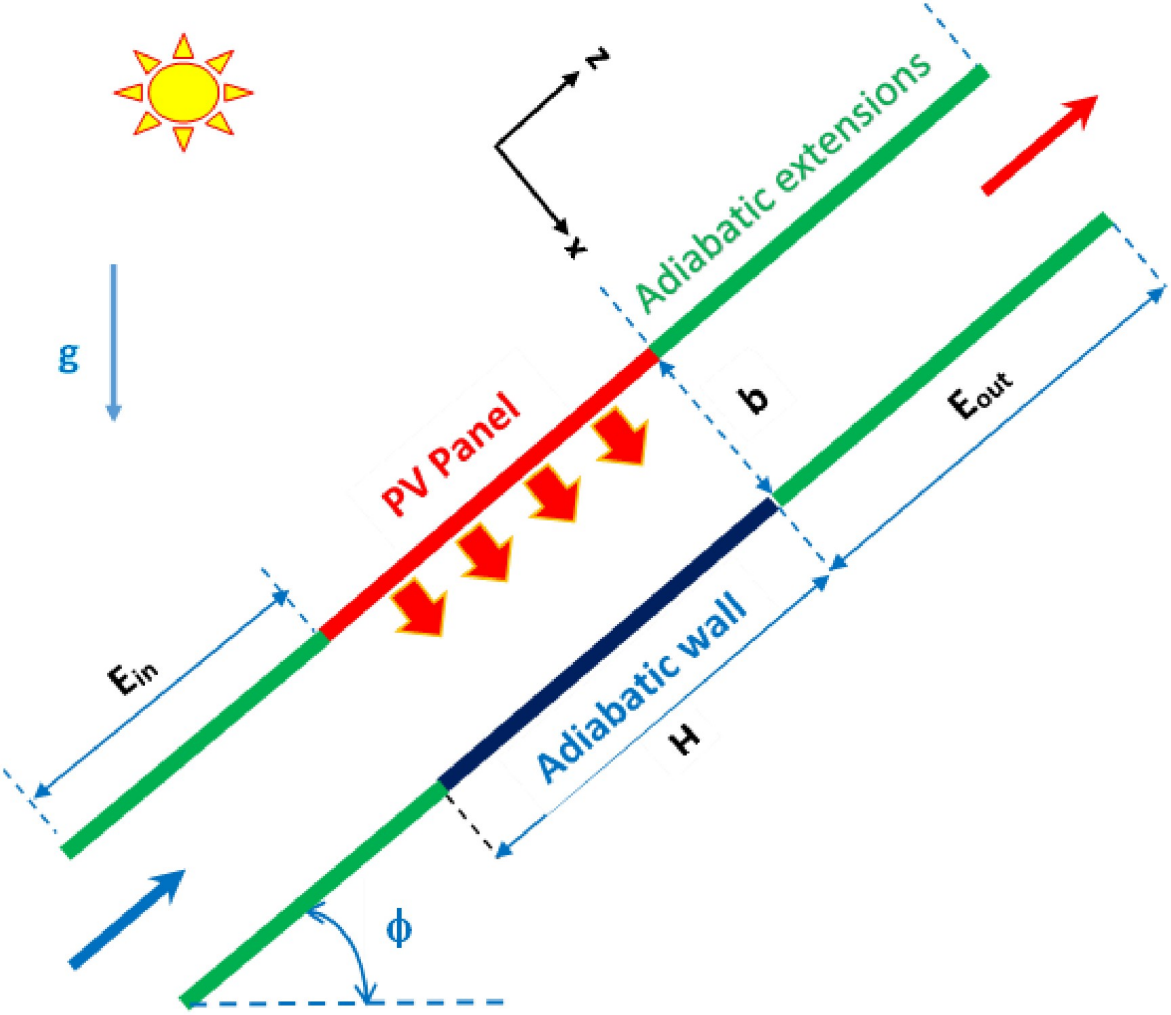
Geometrical configuration of the inclined channel with adiabatic extensions.

The studied configuration is illustrated schematically in Fig 1, with an inclined, open channel formed by two parallel plates in which air can circulate freely. The photovoltaic panel forms the upper wall of the channel, while the lower part is formed by an adiabatic plate of equal length *H*. The channel is inclined to the horizontal plane at an angle ϕ and has an aspect ratio *Ar = b/H* where *b* is the thickness of the air gap behind the photovoltaic panel. The channel is asymmetrically heated by a uniform heat flux *q* applied on its upper surface. Air enters the channel from below at ambient temperature. It flows into the channel under the effect of buoyancy, cooling the back of the photovoltaic module. Adiabatic extensions of lengths E_in_ and E_out_ can be added to the inlet and outlet of the simple channel.

The airflow through the channel is governed by the time-dependent 2D Navier-Stokes and energy equations for an incompressible fluid with constant properties. Fluid motion is assumed to be laminar and two-dimensional.

Using the Boussinesq approximation, the conservation equations of mass, momentum and energy in non-dimensional form are given by [26–29]:

$$\frac{\partial \text{U}}{\partial \text{X}}+\frac{\partial \text{W}}{\partial \text{Z}}=0$$ (1)

$$\frac{\partial \text{U}}{\partial \text{t}}+\text{U}\frac{\partial \text{U}}{\partial \text{X}}+\text{W}\frac{\partial \text{U}}{\partial \text{Z}}=-\frac{\partial \text{P}}{\partial \text{X}}+Pr\left(\frac{\partial^{2}\text{U}}{\partial {\text{X}}^{2}}+\;\frac{\partial^{2}\text{U}}{\partial {\text{Z}}^{2}}\right)+\text{Pr}Ra\;cos\varphi \;T$$ (2)

$$\frac{\partial \text{W}}{\partial \text{t}}+\text{U}\frac{\partial \text{W}}{\partial \text{X}}+\text{W}\frac{\partial \text{W}}{\partial \text{Z}}=-\frac{\partial \text{P}}{\partial \text{Z}}+Pr\left(\frac{\partial^{2}\text{W}}{\partial {\text{X}}^{2}}+\;\frac{\partial^{2}\text{W}}{\partial {\text{Z}}^{2}}\right)+\;\text{Pr}Ra\;sin\varphi \;T$$ (3)

$$\frac{\partial \text{T}}{\partial \text{t}}+\text{U}\frac{\partial \text{T}}{\partial \text{X}}+\text{W}\frac{\partial \text{T}}{\partial \text{Z}}=\left(\frac{\partial^{2}\text{T}}{\partial {\text{X}}^{2}}+\;\frac{\partial^{2}\text{T}}{\partial {\text{Z}}^{2}}\right)$$ (4)

Dimensionless variables are defined as follows:

$$\text{X}=\frac{\text{x}}{\text{b}};\;\text{Z}=\frac{\text{z}}{\text{b}};\;\text{t}=\frac{\alpha \tau }{b^{2}};\;P=\frac{\left(P+\rho gz\right)b^{2}}{\rho \alpha^{2}};\;U=\frac{\text{u}b}{\alpha };\;W=\frac{\text{w}b}{\alpha };\;\text{T}=\frac{\theta -\theta_{\infty }}{\Delta T};\;\Delta T=\text{q}b/\lambda$$

The dimensionless numbers appearing in the governing equations are the Rayleigh number $a=\;\frac{g\beta qb^{4}}{\lambda \alpha \nu }$, the Prandtl number $Pr=\frac{\nu }{\alpha }$ and the modified Rayleigh number Ra* = *Ra* (*b/H*).

The dimensionless number Ra* is the product of the aspect ratio *A*_*r*_ = *b/H* and the Rayleigh number *Ra* and it is the relevant parameter for studying natural convection in heated open channels.

The computational domain is restricted to the channel geometry and the following boundary conditions are imposed:

At the walls, the no-slip boundary condition is applied (*U* = *W* = 0). On the heated wall, a uniform heat flux q is imposed leading to $\frac{\partial T}{\partial X}=-1$ in dimensionless form and along the adiabatic wall $\frac{\partial T}{\partial X}=0$ is assumed.

At the open boundaries (the entrance and exit of the channel),

At the entry of the channel:

$$\;\frac{\partial W}{\partial Z}=0\;;U=0\;;T=0;\;P=-0.5{\left\{\int_{0}^{1}W\left(X,0\right)dX\right\}}^{2}$$

At the exit of the channel:

$$\frac{\partial W}{\partial Z}=\frac{\partial U}{\partial Z}=0\;;\;\frac{\partial T}{\partial Z}=0\;;P=0\;\;\text{If}\;W\geq 0$$

Else

$$\frac{\partial W}{\partial Z}=0\;;U=0\;;T=0;\;P=-0.5\frac{1}{\text{L}}{\left\{\int_{\text{L}}\;W\left(X,0\right)dX\right\}}^{2}$$

L is the length of the portion of the outlet section where the air enters the channel.

The mass flow rate induced by buoyancy is defined as follows:


$$M=\int_{0}^{1}W\left(X,0\right)dX$$

The local Nusselt number along the heated wall is given by:

$$Nu\left(Z\right)=\frac{h\left(Z\right)b}{\lambda }=\frac{1}{T_{w}\left(Z\right)}\;\;\;\;\;\left({\text{T}}_{\text{w}}\;\text{is}\;\text{the}\;\text{wall}\;\text{temperature}\right)$$

The average Nusselt number at heated wall is then defined by:

$$Nu_{a}=\frac{1}{H}\int_{0}^{\text{H}}Nu\left(0,Z\right)dZ$$

## 3. Numerical resolution

The conservation equations are solved using software developed by LIMSI (CNRS FRANCE) for the integration of the time-dependent Navier-Stokes equations. Various types of problems have been solved by this software [27–31].

The Navier-Stokes Eqs (1–4) written in primitive variables are discretized in space by the finite volume method, whereas a time-splitting algorithm is used for temporal integration to decouple velocity and pressure. If all quantities are assumed to be known at time *n*. Δt, the values of the variables at the next time step (*n* + 1) Δt are determined as follows:

First, using a second-order time scheme, an intermediate velocity field V* is determined. For this purpose, the diffusion terms are discretized by a second order backward Euler scheme and the nonlinear terms are processed by the second order Adams-Bashforth scheme [31]. At this point, the discretization is as follows:

$$\frac{3V^{*}-4V^{n}+V^{n+1}}{2\Delta t}+2{\left(V.\nabla V\right)}^{n}-{\left(V.\nabla V\right)}^{n-1}=-\nabla P^{n}+Pr\Delta V^{*}$$ (5)

Then, using Helmholtz’s decomposition theorem, the intermediate velocity field is projected onto the subspace of divergence free vector field:

$$V^{n+1}-V^{*}=-\frac{2\Delta t}{3}\nabla \left(P^{n+1}-P^{n}\right)$$ (6)

When applying the divergence operator to the previous expression, we obtain a Poisson-type equation for the incremental pressure:

$$\Delta \Psi =\frac{3}{2\Delta t}\nabla V^{*}\;\Psi =P^{n+1}-P^{n}$$ (7)

Finally, the last equation is solved by a multigrid method that accelerates the convergence of the basic iterative method by using a hierarchy of discretization.

Next, the new quantities at time (*n* + 1). Δt are given by:

$$\left\{\begin{array}{c} P^{n+1}=\Psi \;+\;P^{n}\\ V^{n+1}=V^{*}-\frac{2\Delta t}{3}\nabla \Psi \end{array}\right.$$ (8)

Spatial discretization uses a second order upwind scheme for the convective terms and a centered scheme for the diffusive terms.

The calculations were carried out on a 2D computational domain limited to the space bounded by the channel walls. This domain is defined by [0, *X*_*m*_] * [0, *Z*_*m*_] where X_m_ = 1 and Z_m_ = H/b. The domain is simple and has no singularities. Consequently, refinement is only used close to the walls. The spatial discretization used consists of a uniform mesh in the flow direction and a non-uniform mesh in geometric progression in the normal direction. The finest meshes are located close to the walls.

A mesh independence study was conducted to guarantee the accuracy of the numerical results for the vertical channel. Calculations were made with three gridline systems, namely 66*130, 130*258 and 258*514 with Ra* = 10^5^. The changes in the mesh size give errors of less than 1.5% for relevant parameters such as the average Nusselt number and the mass flow rate. Based on the test results, the mesh of 130*258 is chosen for all simulations. Throughout this work, the time step is set at Δt = 10^−5^, close to the limit of stability and the maximum CFL number is around 0.5, close to the walls.

## 4. Code validation

**Fig 2:**
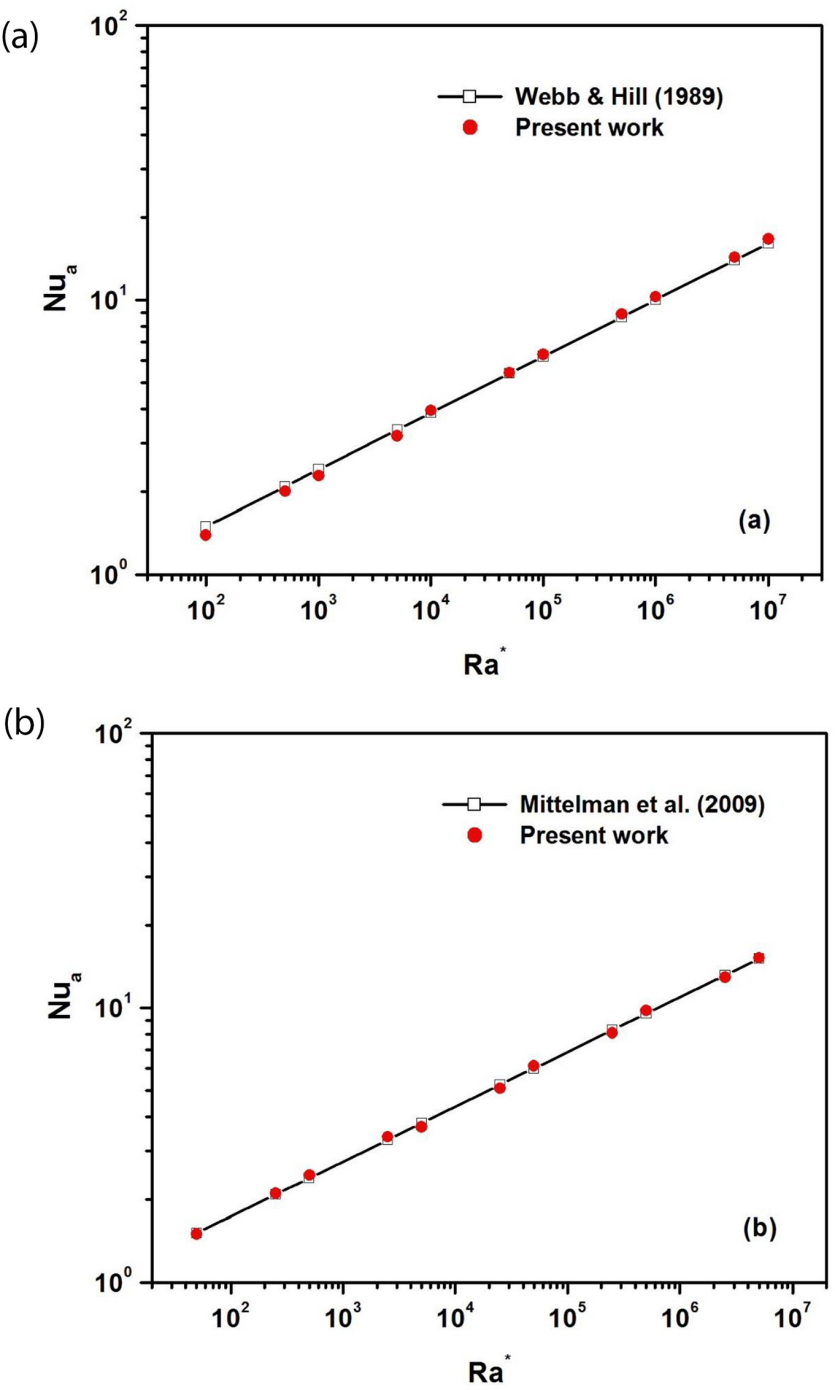
**a:** Average Nusselt number versus the modified Rayleigh number in the case of a vertical channel (A_r_ = b/H = 1/5 and ϕ = 90°). **b:** Average Nusselt number versus the modified Rayleigh number in the case of an inclined channel (A_r_ = b/H = 1/30 and ϕ = 30°).

To validate our numerical model, comparisons with results available in the literature were performed. The first validation test relates to the Webb and Hill experiment [32] for a vertical asymmetrically heated channel with an aspect ratio *A*_*r*_ = *b/H* = 1/5. The variation of the average Nusselt number with the modified Rayleigh number in the case of the vertical channel (ϕ = 90°) is displayed in Fig 2a. The computed values of the average Nusselt number in this simulation are in good agreement with the correlation proposed in [32].

The second validation test uses the numerical results presented by Mittelman et al. relating to the passive cooling of inclined photovoltaic panels tilted by an angle (ϕ = 30°) and with an aspect ratio *A*_*r*_ = *b/H* = 1/20 [33]. Fig 2b depicts the variation of the average Nusselt number with the modified Rayleigh number in the case of the inclined channel (Ar = b/H = 1/30 and ϕ = 30°). As can be seen, our results are in perfect agreement with the correlations given by Mittelman et al. [33].

## 5. Results and discussion

Numerical modeling of natural convection flows in open channels is simple, but it is difficult even in the 2D case, especially when the computational domain is limited to the channel space. The boundary conditions at the channel entrance are unknown and only the exit conditions can be imposed. This difficulty is generally overcome by using homogeneous boundary conditions of the Neumann type. The exact determination of the boundary conditions to be imposed at the entrance remains an open problem because the flow is generated by the hot source located downstream of the channel entrance.

The modified Rayleigh number Ra* is the most relevant parameter for characterizing the flow regime in heated open channels. It should be noted that the flow regime in the asymmetrically heated channel is laminar for Ra* ≤ 10^6^ and turbulent for Ra* ≥ 10^7^.

The effect of inclination on airflow and heat transfer in channels with and without adiabatic extensions is studied in a laminar regime. In the following, the channel configuration without adiabatic extensions will be referred to as the reference case. The study covers a wide range of tilt angles (15° ≤ ϕ ≤ 90°) and modified Rayleigh numbers (10^2^≤ Ra* ≤10^6^).

Preliminary calculations are first performed on the channel without extensions to predict the critical aspect ratio that minimizes overheating of the photovoltaic panel for different tilt angles and various modified Rayleigh numbers.

### 5.1 Channel without extensions

In this section, we examine the case of the channel without extensions (the reference case) before analyzing the effects of extensions on channel performance.

#### 5.1.1 Effect of aspect ratio

To study the effects of the aspect ratio Ar on natural convection in inclined channels, Ar was varied from Ar = 1/20 = 0.05 to Ar = 1/5 = 0.2 for different angles of inclination.

As the length of the channel is fixed, varying the spacing between the walls results in a variation of the aspect ratio. Reducing the aspect ratio means reducing the air gap behind the photovoltaic panel. This section aims to determine the critical aspect ratio that minimizes overheating. The critical aspect ratio can be defined as the value of the aspect ratio at which the mass flow rate in the channel and the maximum temperature of the PV panel begin to stabilize. This approach was used by Gan (2009) [34] to determine the critical air gaps for building-integrated photovoltaics. Determining the critical value of the aspect ratio means determining the minimum channel width that prevents the photovoltaic panel from overheating.

**Fig 3:**
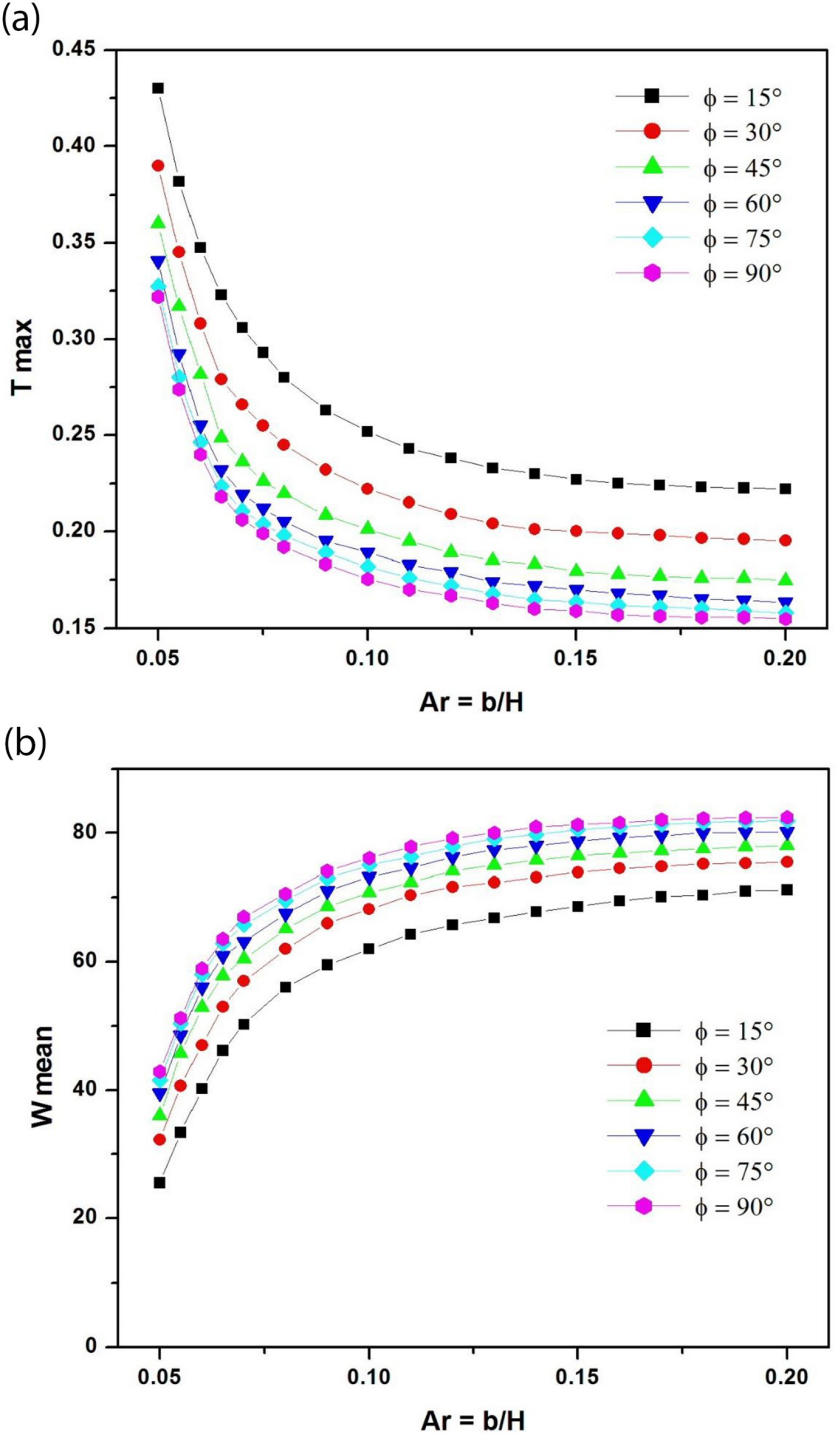
**a**: Maximum dimensionless PV module temperature as a function of channel aspect ratio for different tilt angles at Ra* = 10^5^ (channel without extensions). **b**: Dimensionless mean velocity as a function of channel aspect ratio for different tilt angles at Ra* = 10^5^ (channel without extensions).

Fig 3a and 3b show respectively the variation of dimensionless maximum PV temperature and mean air velocity as a function of channel aspect ratio for tilt angles ranging from 15° to 90° at Ra* = 10^5^. It should be noted that for the same aspect ratio, the maximum temperature of the PV panel decreases as the angle of inclination increases (closer to the vertical position). This result can be explained by the increase in air flow rate as the channel inclination tends towards the vertical position (Fig 3b). It can also be seen that as the aspect ratio decreases, the maximum temperature of the PV panel remains stable until a critical value is reached when the temperature rises significantly to high values indicating high overheating at low aspect ratios. This behavior is due to the decrease in air flow rate for low aspect ratios and consequently for low air gaps (Fig 3b). From these two curves, we can estimate the critical aspect ratio to be around 0.1 (Ar_c_ = 1/10). This means that the minimum spacing between the channel walls must be H/10 a tenth of the panel length H. In the following, the channel aspect ratio will be fixed at Ar = 1/8 above the critical value.

#### 5.1.2 Fluid flow and heat transfer characteristics

**Fig 4:**
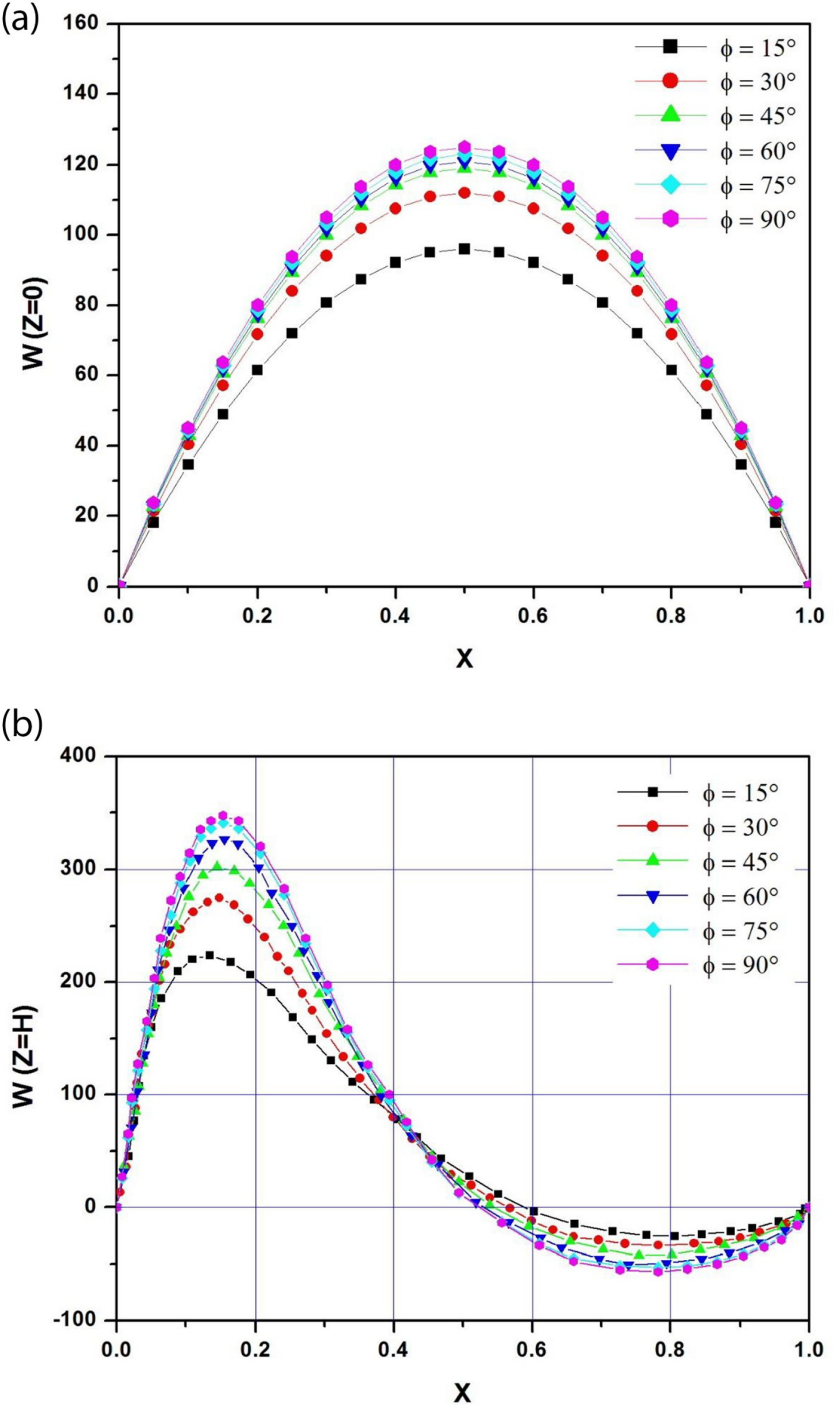
**a**: Velocity profiles at the channel inlet for different tilt angles (A_r_ = b/H = 1/8; Ra* = 10^5^). **b**: Velocity profiles at the channel outlet for different tilt angles (A_r_ = b/H = 1/8; Ra* = 10^5^).

To study the flow structure inside the simple channel, the velocity profiles at the channel inlet and outlet are analyzed for different angles of inclination. Fig 4a and 4b depict respectively the velocity profiles at the entrance and exit of the channel for pitch angles ranging between 15° and 90° for a modified Rayleigh number Ra* = 10^5^ and an aspect ratio Ar = 1/8. Velocity profiles at the channel inlet are of the fully developed parabolic type for all inclinations. Peak velocity is symmetrical and increases with the inclination angle. The velocity profiles are very close for 45° ≤ ϕ ≤ 90°, while a remarkable drop is observed for ϕ = 15°.

At the exit of the channel, the velocity profiles are of the boundary layer type, with a strong asymmetry highlighted by the acceleration of the fluid behind the PV panel. A reversal zone, characterized by negative velocities, partially occupies the channel outlet near the lower adiabatic wall. Its size increases with the tilt angle. The peak velocity also increases as the angle of inclination increases and gets closer to the photovoltaic panel, thus reducing the thickness of the boundary layer.

The maximum velocity at the channel inlet and outlet increases with the angle of inclination. An inversion zone, characterized by negative velocities, partially occupies the channel outlet near the lower adiabatic wall. Its size increases with the tilt angle.

**Fig 5:**
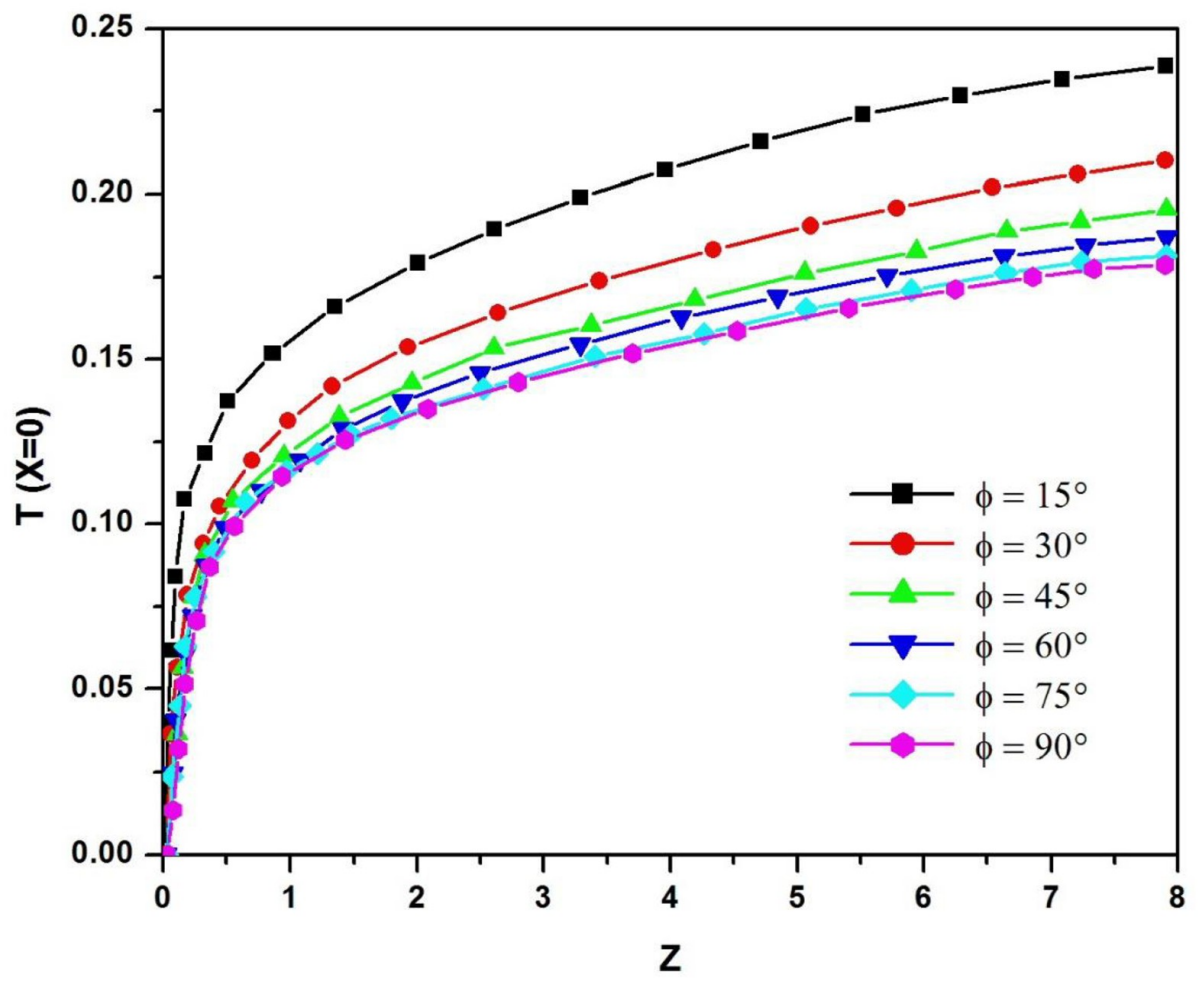
Dimensionless temperature profiles along the upper channel wall for different tilt angles (A_r_ = b/H = 1/8; Ra* = 10^5^).

To study heat exchange inside the channel, we examine the temperature profiles along the heated wall for different angles of inclination. Fig 5 shows dimensionless temperature profiles along the top wall of the channel for different angles of inclination between 15° and 90° for a modified Rayleigh number Ra* = 10^5^ and an aspect ratio Ar = 1/8. These profiles take the form of a charge curve. At the inlet, the dimensionless temperature is zero, then increases rapidly until it reaches the maximum temperature at the channel outlet, meaning that the overheating zone is at the upper end of the PV panel.

**Fig 6:**
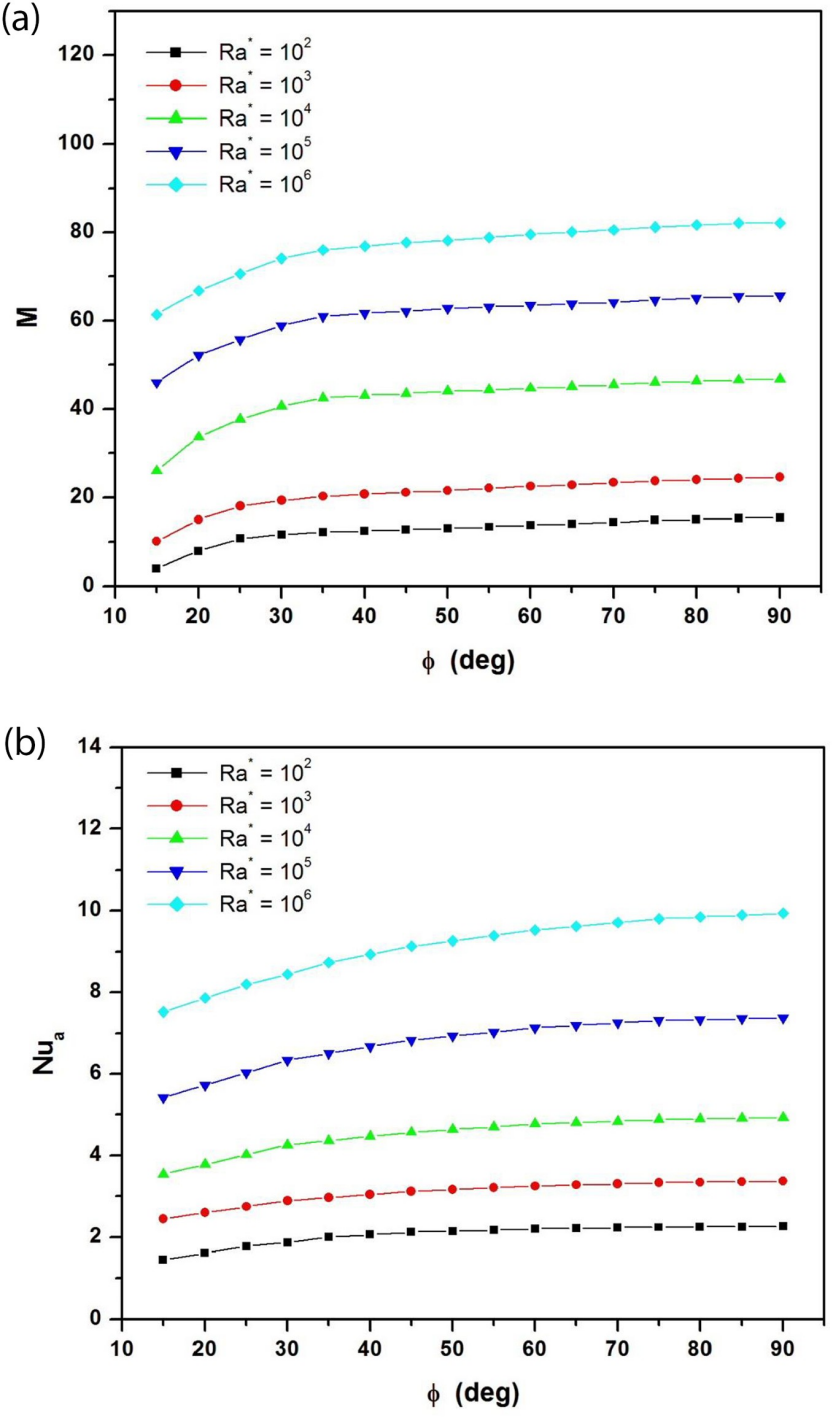
**a**: Dimensionless mass flow rate as a function of tilt angles for different modified Rayleigh numbers (A_r_ = b/H = 1/8). **b**: Average Nusselt number as a function of tilt angles for different modified Rayleigh numbers (A_r_ = b/H = 1/8).

To quantify heat and mass transfer within the channel, we examine the variations of average Nusselt number and mass flow rate as a function of tilt angle. Fig 6a and 6b show respectively the dimensionless mass flow rate and the average Nusselt number versus the tilt angles for different modified Rayleigh numbers ranging from Ra* = 10^2^ to Ra* = 10^6^ when the aspect ratio is fixed at (Ar = 1/8). The mass flow rate increases with the modified Rayleigh number and with the angle of inclination. The increase is particularly marked at low angles of inclination. It should also be noted that the average Nusselt number increases with the modified Rayleigh number and with the angle of inclination. This means that heat exchange intensifies as the inclination of the channel increases and approaches the vertical position. But for tilt angles below 30°, the rate of heat transfer remains particularly low.

### 5.2 Channel with extensions

#### 5.2.1 Effects of adiabatic extensions

In previous numerical and experimental studies, some authors have introduced adiabatic extensions at the entrance and exit of the channel. The reasons for this are various: stabilization of the flow, minimization of radiation losses or simply improvement of the thermal and hydrodynamic performance of the open channel. The purpose of this paragraph is to verify the usefulness of adding extensions to the input or output of the channel to improve its performance.

To enhance heat transfer and buoyancy-induced mass flow, adiabatic extensions are added to the inlet and outlet of the simple channel. Fig 1 shows schematically the geometric configuration of the inclined channel with adiabatic extensions of lengths E_in_ and E_out_. In the following, the aspect ratio of the simple channel is fixed at (Ar = 1/8). Next, extensions of lengths H/2 and H are added at either the inlet or outlet of the channel to test their usefulness.

**Fig 7:**
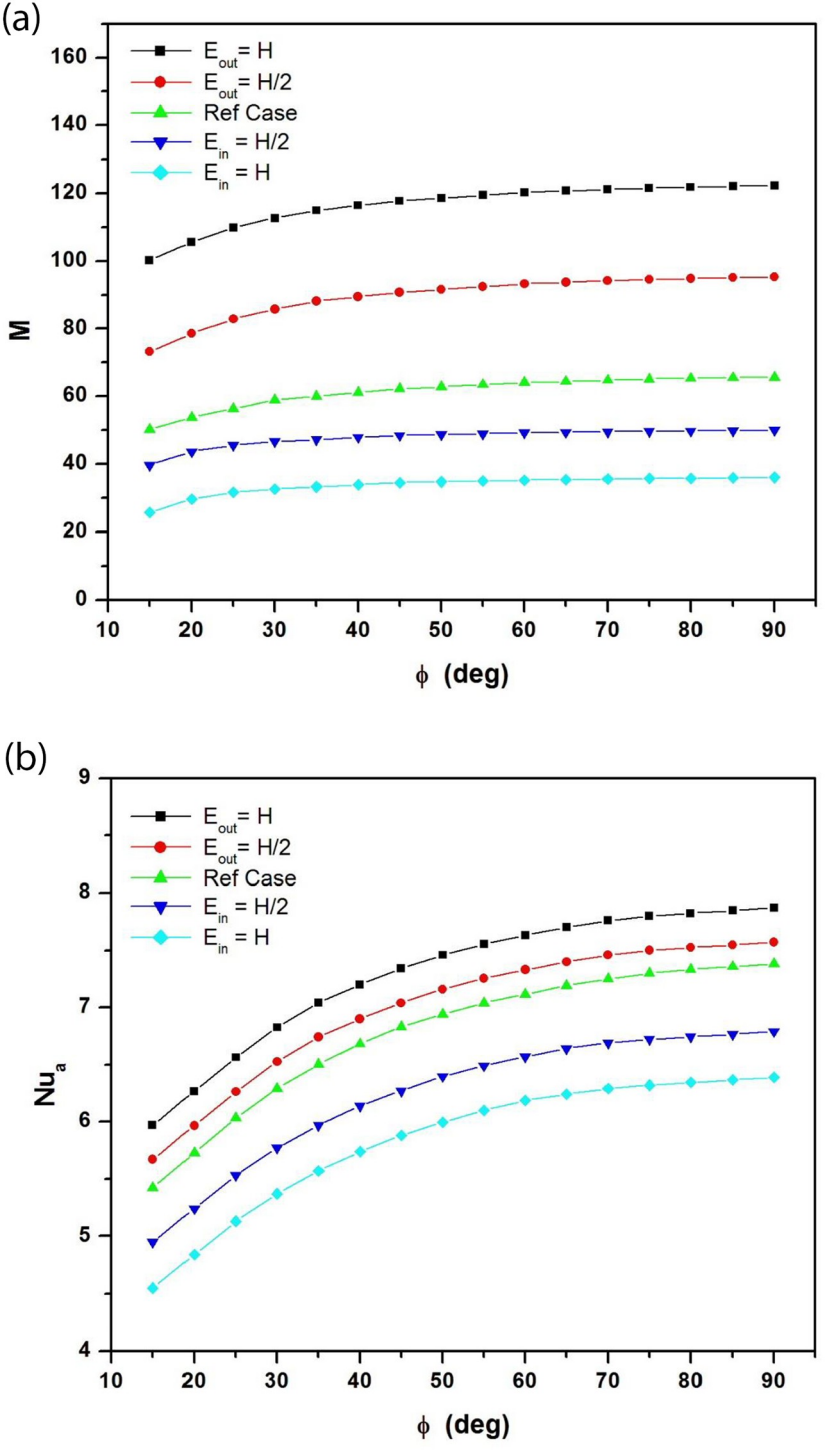
**a**: Dimensionless mass flow rate as a function of tilt angles for different adiabatic extensions at the channel inlet and outlet (A_r_ = b/H = 1/8; Ra* = 10^5^). **b**: Average Nusselt number as a function of tilt angles for different adiabatic extensions at the channel inlet and outlet (A_r_ = b/H = 1/8; Ra* = 10^5^).

Fig 7a and 7b respectively show the variation of the dimensionless mass flow rate M and the average Nusselt number Nu_a_ as a function of the angles of inclination for different adiabatic extensions at the entrance and at the exit of the channel when the modified Rayleigh number is equal to Ra* = 10^5^.

The introduction of the extension at the channel inlet does not improve mass flow rate and heat exchange for all tilt angles (15°≤ ϕ ≤ 90°) considered in the study. Increasing the length of these extensions even inhibits airflow in the channel and heat transfer at the rear of the photovoltaic panel. For example, when the modified Rayleigh number is Ra* = 10^5^ and the inlet extension is of length E_in_ = H, the reduction in mass flow rate is greater than 45% for all tilt angles, resulting in a 14% reduction in average Nusselt number for the same conditions. On the other hand, the addition of extensions at the channel outlet improves both the mass flow rate and Nusselt number for all angles of inclination. Extending the length of these extensions even boosts airflow in the channel and heat transfer rate at the heated wall. For example, when the modified Rayleigh number is Ra* = 10^5^ and the outlet extension length is E_out_ = H, the enhancement in mass flow rate M is over 47% for all tilt angles, resulting in a 7% increase in heat transfer rate for the same conditions. In conclusion, extensions at the channel inlet should be avoided and only extensions at the channel outlet should be introduced to improve both mass flow rate and average Nusselt number.

To explain the mechanisms behind the negative effects of inlet extensions on airflow and heat transfer, we may suspect a loss of chimney draught power or the formation of a recirculation zone blocking the channel inlet. The first hypothesis is to be rejected, as increasing the length of a chimney always increases its draught due to heat loss. It should also be noted that the second hypothesis is also to be rejected, as all numerical and experimental studies on heated channels have never mentioned the formation of a recirculation zone at the inlet [21, 32–35]. In our opinion, the real reason for the poor performance of inlet extensions is the presence, in this zone, of fresh, dense air that cannot flow by buoyancy because it has not yet been heated. This mass of cold air blocks the entrance of the channel and reduces the airflow through the channel, as well as heat extraction from the PV panel.

#### 5.2.2 Effects of extension length

In this section, we study the effect of varying the extension length at the channel outlet, on the mass flow rate and the heat transfer rate along the heated upper wall. The channel tilt angle is fixed at ϕ = 30°. This choice is dictated by the open-air tests currently underway in the Tabuk region of Saudi Arabia (latitude 30°). These experiments provide interesting details on the actual performance of the system under desert conditions.

**Fig 8:**
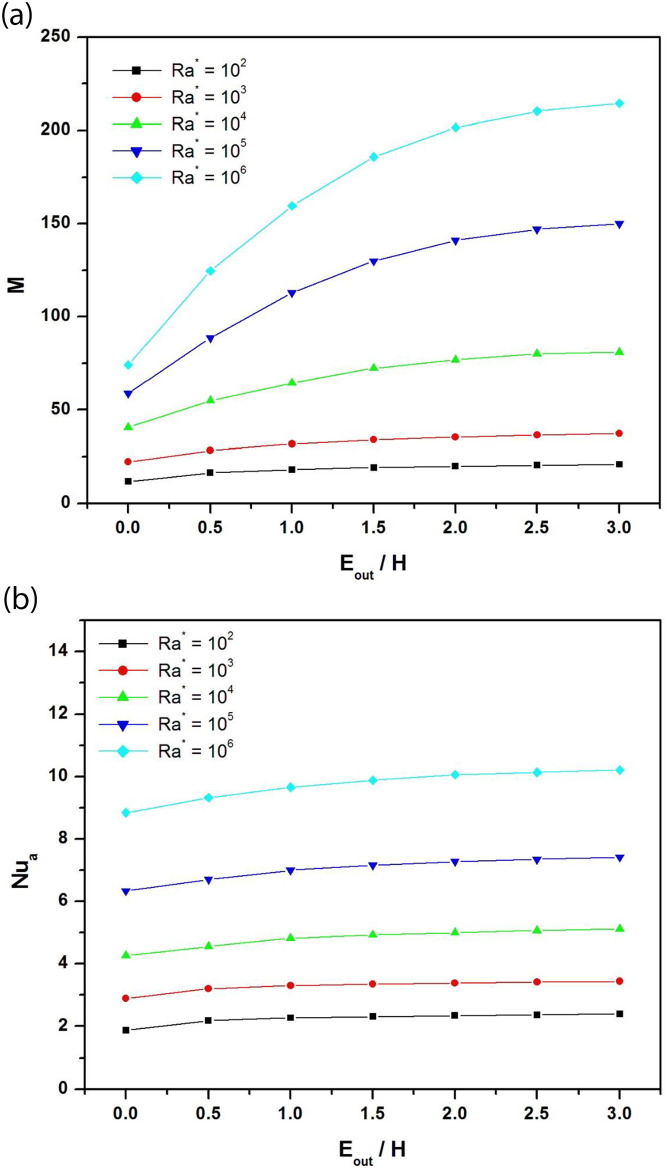
**a**: Dimensionless mass flow rate as a function of normalized adiabatic extension at the channel outlet for different modified Rayleigh numbers (A_r_ = b/H = 1/8; ϕ = 30°). **b**: Average Nusselt number as a function of normalized adiabatic extension at the channel outlet for different modified Rayleigh numbers (A_r_ = b/H = 1/8; ϕ = 30°).

For simulation, the extension length at the channel outlet E_out_ was varied from 0 to 3H. Fig 8a and 8b depict, respectively, the variation of dimensionless mass flow and average Nusselt number as a function of normalized adiabatic extension E_out_/H at the channel outlet for different modified Rayleigh numbers ranging from Ra* = 10^2^ to 10^6^ when (Ar = 1/8; ϕ = 30°).

Interestingly, the mass flow rate and Nusselt number increase with the outlet extension length E_out_ and the modified Rayleigh number Ra*. These increases are significant when the extension length E_out_ exceeds 2H. For the mass flow, the best improvement rates are obtained for Ra* = 10^6^. For example, when the extension length E_out_ increases from 0 to 3H, the mass flow rate increases by 50% at Ra* = 10^4^, by 60% at Ra* = 10^5^ and by 65% at Ra* = 10^6^. However, the best improvement rates of the average Nusselt number are obtained for low Rayleigh numbers. Indeed, when the length of extension goes from 0 to 3H, the Nu_a_ improvement rate is 21.7% at Ra* = 10^2^, 16.8% at Ra* = 10^4,^ and 13.4% at Ra* = 10^6^.

**Fig 9:**
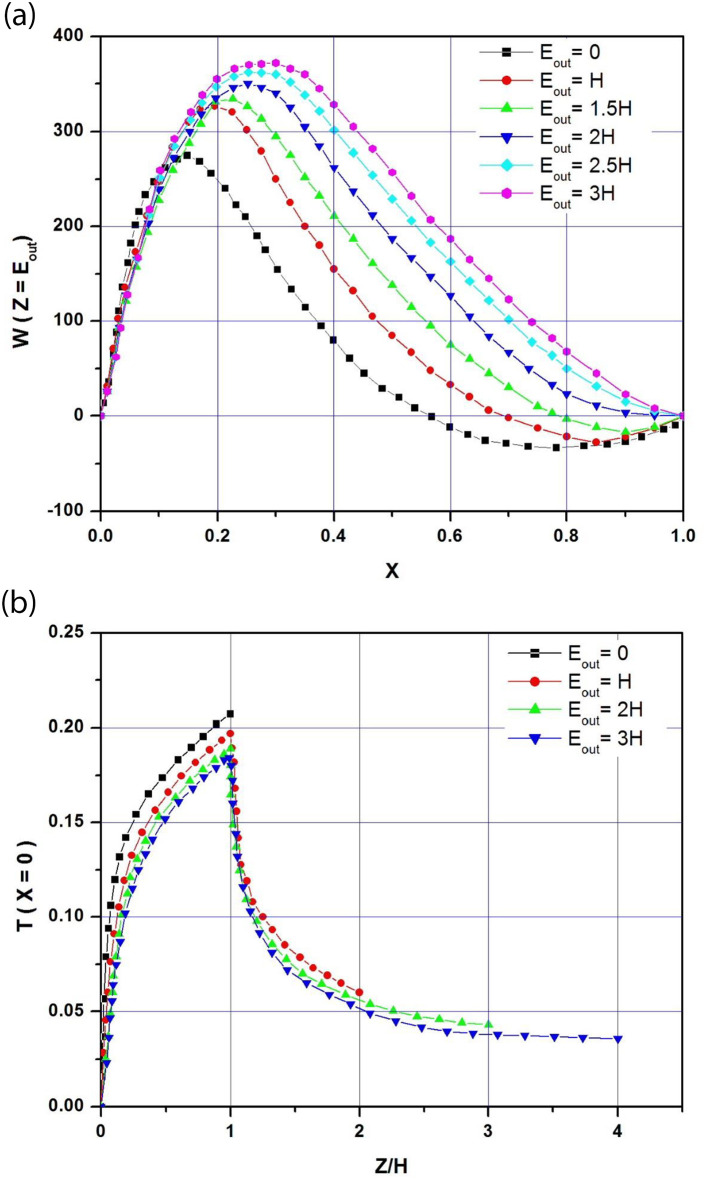
**a**: Channel exit velocity profiles for different adiabatic extension E_out_ (A_r_ = b/H = 1/8; Ra* = 10^5^; ϕ = 30°). **b**: Dimensionless temperature profiles along the upper channel wall for different adiabatic extension E_out_ (A_r_ = b/H = 1/8; Ra* = 10^5^; ϕ = 30°).

To analyze the results and explain the performance of long extensions, we study the evolution of thermal and dynamic fields in channels of different lengths. It should be noted that varying Rayleigh numbers and extension lengths lead to complex airflow patterns, particularly at the channel outlet. Fig 9a shows channel exit velocity profiles for different E_out_ extension lengths ranging from 0 to 3H (Ar = 1/8; Ra* = 10^5^; ϕ = 30°). For the reference case (E_out_ = 0), the velocity profile at the outlet of the channel is of the boundary layer type, with a strong asymmetry and a sharp peak due to the acceleration of the fluid near the heated wall. Negative velocities are observed near the adiabatic wall. In this area, air enters the channel in the opposite direction through the outlet section, creating an inversion zone. This recirculation area partially blocks the channel outlet, reducing the airflow rate. As the length of the extensions increases, the chimney draft intensifies the mass flow rate increases, and the peak velocity shifts towards the channel axis. The inversion zone, corresponding to negative velocities, narrows as the extensions lengthen until it disappears for E_out_ = 2H. The disappearance of the reversed flow, which partially blocks the exit of the channel, frees the airflow and improves the mass flow rate. As the length of the extensions increases, the velocity profiles gradually change from the boundary layer type for E_out_ = 0 to the asymmetric Poiseuille type for E_out_>2H, characterized by a broad peak that tends to approach the channel axis. As noted above, the experimental studies of flows in asymmetrically heated channels, with or without extension, have never shown the existence of a recirculation zone at the channel inlet. However, reverse flow does occur at the channel outlet, close to the adiabatic wall under certain conditions. As the length of the outlet extensions increases, the size of this vortex decreases until it disappears for an extension length twice that of the panel. This can be explained by the increase in the chimney’s draught power as a result of the increased channel length.

Dimensionless temperature profiles along the top wall of the channel are depicted in Fig 9b for adiabatic extensions with lengths ranging from 0 to 3H when Ar = 1/8; Ra* = 10^5^ and ϕ = 30°. These profiles have the same shape as the charge and discharge curves. At the entrance of the channel, the dimensionless temperature is zero then it increases rapidly along the heated wall until it reaches its maximum value at the upper end of the PV panel. Then, the temperature decreases exponentially along the adiabatic extension until it reaches its minimum at the exit of the channel. As the length of the extensions increases, the airflow intensifies and the temperature of the PV module drops, thus avoiding the risk of overheating and improving the electrical performance of the PV panel. For example, as the length of the extension increases from 0 to 3H, the maximum temperature decreases by 4.8% for E_out_ = H, 8.7% for E_out_ = 2H and 11.1% for E_out_ = 3H.

The study showed that the reverse flow which is partially blocking the channel outlet disappears as soon as the length of the extensions exceeds twice that of the panel. Beyond this length, improvements in mass flow and heat transfer rate are not significant. Clearly, increasing the size of the extensions will be accompanied by higher purchase and installation costs. What’s more, if the space reserved for panel installation is limited, like the roof of a building for example, you can’t afford to use extensions that are too long. For this reason, using extensions twice the length of the panel is a good compromise between technical and economic constraints. This critical length also avoids the risk of shading other PV modules due to the excessive size of the extensions, and also avoids the use of cumbersome mounting structures to hold them in place.

The proposed system is easy to install, economical, silent, energy-efficient and maintenance-free. It protects the photovoltaic panel from overheating, increasing its efficiency and lifespan. This cooling technique is particularly suited to arid regions where the climate is hot and water scarce. However, in these arid regions, the performance of the proposed system can be influenced by extreme weather conditions, such as intense solar radiation, high ambient temperatures, external winds and sandstorms. Dust accumulation is a major challenge to the proper operation of the photovoltaic panel and passive cooling system. Regular maintenance is necessary to ensure efficiency.

## 6. Conclusion

A numerical study was carried out on the use of natural convection to cool a photovoltaic panel using an inclined chimney at the rear. Validated through numerical simulations, the model considers modified Rayleigh numbers, tilt angles, and channel aspect ratios to evaluate the performance of the simple channel. A critical aspect ratio of 0.1 is identified to prevent PV module overheating, emphasizing the need for an air gap at least one-tenth of the panel length. The analysis of the simple channel’s flow reveals a complex structure influenced by inclination and Rayleigh number. Increasing Rayleigh number and tilt angle enhances airflow, intensifying heat exchange, and expediting panel cooling. Adiabatic extensions at the channel’s entrance and exit are introduced to improve chimney draught and cooling. Inlet extensions hinder airflow, while outlet extensions significantly enhance mass flow and Nusselt number. Critical findings show a 47% increase in mass flow and a 7% rise in heat transfer rate when outlet extensions equal panel height. A critical extension length of 2H is identified, leading to improved heat exchange, mass flow rate, and a reduction in maximum temperature. It was found that increasing the length of the extensions from 0 to 3H improved the mass flow rate by 65%, the average Nusselt number by 13.4%, and led to an 11% drop in maximum temperature, when Ra* = 10^6^. The proposed system, easy to install and cost-effective, safeguards the photovoltaic panel from overheating, enhancing efficiency and lifespan, making it ideal for arid regions with hot climates and water scarcity. However, the performance of the proposed system can be influenced by extreme weather conditions, such as intense solar radiation, high ambient temperatures, external winds, sandstorms and dust accumulation.

## Supporting information

S1 Fig OPJ: Origin data for Fig 2a. (OPJ)

S2 Fig OPJ: Origin data for Fig 2b. (OPJ)

S3 Fig OPJ: Origin data for Fig 3a. (OPJ)

S4 Fig OPJ: Origin data for Fig 3b. (OPJ)

S5 Fig OPJ: Origin data for Fig 4a. (OPJ)

S6 Fig OPJ: Origin data for Fig 4b. (OPJ)

S7 Fig OPJ: Origin data for Fig 5. (OPJ)

S8 Fig OPJ: Origin data for Fig 6a. (OPJ)

S9 Fig OPJ: Origin data for Fig 6b. (OPJ)

S10 Fig OPJ: Origin data for Fig 7a. (OPJ)

S11 Fig OPJ: Origin data for Fig 7b. (OPJ)

S12 Fig OPJ: Origin data for Fig 8a. (OPJ)

S13 Fig OPJ: Origin data for Fig 8b. (OPJ)

S14 Fig OPJ: Origin data for Fig 9a. (OPJ)

S15 Fig OPJ: Origin data for Fig 9b. (OPJ)

## References

[1] Pourasl H. H., Barenji R. V., Khojastehnezhad V. M., (2023), Solar energy status in the world: A comprehensive review, Energy Reports, Vol.10, 2023, 3474–3493. doi: 10.1016/j.egyr.2023.10.022

[2] Du Y., Fell C. J., Duck B., Chen D., Liffman K., Zhang Y., et al, (2016), Evaluation of photovoltaic panel temperature in realistic scenarios, Energy Conversion and Management, Vol.108, 2016,60–67, doi: 10.1016/j.enconman.2015.10.065

[3] Sheik M. S., Kakati P., Dandotiya D., Ravi U.M, Ramesh C S, (2022), A comprehensive review on various cooling techniques to decrease an operating temperature of solar photovoltaic panels, Energy Nexus, Vol.8, 2022,100161, doi: 10.1016/j.nexus.2022.100161

[4] Badi N; Alghamdi S.A.; El-Hageen H.M.; Albalawi H.; (2023). Onsite enhancement of REEEC solar photovoltaic performance through PCM cooling technique. PLOS ONE, 18, 2023, e0281391. doi: 10.1371/journal.pone.0281391 36897855 PMC10004609

[5] Shukla A, Kant K, Sharma A, Biwole PH. (2017). Cooling methodologies of photovoltaic module for enhancing electrical efficiency: A review. Sol Energy Mater Sol Cells 2017;160:275–86.

[6] Hasanuzzaman M., Abma M., Islam M.M., Pandey A.K., Rahim N.A. (2016). Global advancement of cooling technologies for PV systems: A review. Sol Energy, 2016;137:25–45.

[7] Dwivedi P., Sudhakar K., Soni A., Solomin E., Kirpichnikova I. (2020). Advanced cooling techniques of PV modules: A state of art, Case Stud. Therm. Eng., 21 (2020), Article 100674.

[8] Maleki A., Haghighi A., El Haj Assad M., Mahariq I., Nazari M. A. (2020). A review on the approaches employed for cooling PV cells, Solar Energy 2020;209:170–185.

[9] Jiao C.; Li Z. (2023), An Updated Review of Solar Cooling Systems Driven by Photovoltaic–Thermal Collectors. Energies 2023, 16, 5331. doi: 10.3390/en16145331

[10] Brinkworth B.J, Cross B.M, Marshall R.H, Yang H., (1997).Thermal regulation of photovoltaic cladding, Solar Energy, Vol. 61, Issue 3,1997,169–178.

[11] Tripanagnostopoulos Y, Themelis P. (2010). Natural flow air cooled photovoltaics. AIP Conf Proc. 2010;1203:1013–1018.

[12] Baloch A.B., Bahaidarah H., Gandhidasan P., Al-Sulaiman F., (2015). Experimental and numerical performance analysis of a converging channel heat exchanger for PV cooling, Energy Conversion and Management,Vol. 103,2015,14–27.

[13] Abd-Elhady M.S., Serag Z., Kandil H.A., (2018). An innovative solution to the overheating problem of PV panels, Energy Conversion and Management,Vol.157,2018,452–459.

[14] Glick A., Ali N., Bossuyt J. et al. Utility-scale solar PV performance enhancements through system-level modifications. Sci Rep 10, 10505 (2020). doi: 10.1038/s41598-020-66347-5 32601328 PMC7324397

[15] Naghavi M.S., Esmaeilzadeh A., Singh B., Ang B.C., Yoon T.M., Ong K.S., (2021). Experimental and numerical assessments of underlying natural air movement on PV modules temperature, Solar Energy, Vol. 216,2021,610–622.

[16] Zhou Z., Tkachenko S., Bahl P., Tavener D., de Silva C., Timchenko V., et al, (2022), Passive PV module cooling under free convection through vortex generators, Renewable Energy,Vol.190,2022,319–329.

[17] Sutanto B., Indartono Y. S., Wijayanta A. T., Iacovides H., (2022). Enhancing the performance of floating photovoltaic system by using thermosiphon cooling method: Numerical and experimental analyses, International Journal of Thermal Sciences, Vol.180,2022, 107727.

[18] Fatnassi S., Abidi-Saad A., Bouraoui M. et al. Experimental free convection-induced flow inside an unevenly heated inclined duct in the transient and laminar regimes; application to PV-DSF installations. Heat Mass Transfer 58, 2161–2174 (2022).

[19] Panda S., Panda B., Jena C., Nanda L., Pradhan A., (2023). Investigating the similarities and differences between front and back surface cooling for PV panels, Materials Today: Proceedings, Vol.74, Part 2, 2023,358–363.

[20] El Kharaz H., Khallaki K., Kadiri M. S., Choukairy K., (2024), A numerical analysis of air flow topology within a vertical channel attached behind photovoltaic panel, International Journal of Heat and Mass Transfer, Volume 223, 2024,125254. doi: 10.1016/j.ijheatmasstransfer.2024.125254

[21] Campo A., Manca O., Morrone B., (1999), Numerical analysis of partially heated vertical parallel plates in natural convective cooling, Numerical Heat Transfer A, 36 (1999) 129–151. doi: 10.1080/104077899274813

[22] Bassiouny R., Korah N., (2009), Effect of solar chimney inclination angle on space flow pattern and ventilation rate, Energy Build. 41 (2009) 190–196.

[23] Roy K., Das B., Pathak K. K., Giri A. (2022). Thermo-Hydraulic Analysis of Slightly Inclined Finned Channel under Natural Convection, Journal of Applied Fluid Mechanics, 15(4), pp. 985–998. doi: 10.47176/jafm.15.04.33307

[24] Roy K., Das B., (2020), Convective heat transfer from an inclined isothermal fin array: A computational study, Thermal Science and Engineering Progress, Vol. 17, 2020,100487. doi: 10.1016/j.tsep.2020.100487

[25] Das B., Giri A., (2015), Mixed convective heat transfer from vertical fin array in the presence of vortex generator, International Journal of Heat and Mass Transfer,Vol.82, 2015, 26–41, doi: 10.1016/j.ijheatmasstransfer.2014.11.030

[26] Desrayaud G., Chénier E., Joulin A., Bastide A., Brangeon B., Caltagirone J.P., et al, (2013), Benchmark solutions for natural convection flows in vertical channels submitted to different open boundary conditions, International Journal of Thermal Sciences, Vol.72, 2013, 18–33. doi: 10.1016/j.ijthermalsci.2013.05.003

[27] Taieb S., Laatar A.H., Balti J. (2013). Natural convection in an asymmetrically heated vertical channel with an adiabatic auxiliary plate. International Journal of Thermal Sciences 74:24–36

[28] Nasri Z., Laatar A.H., Balti J. (2015) Natural convection enhancement in an asymmetrically heated channel-chimney system. International Journal of Thermal Sciences 90:122–134.

[29] Nasri Z., Derouich Y., Laatar A.H., Balti J. (2018) Effect of surface radiation on natural convection in an asymmetrically heated channel-chimney system. Heat Mass Transfer 54: 1511.

[30] Derouich Y., Nasri Z., Abide S., Laatar A.H., (2018). Inclination effects on heat transfer by an oscillating square cylinder in channel flow, International Journal of Heat and Mass Transfer,Vol.125,2018,1105–1120.

[31] Laatar A.H., Benahmed M., Belghith A., Le Quéré P., (2002), 2D large eddy simulation of pollutant dispersion around a covered roadway, Journal of Wind Engineering and Industrial Aerodynamics 90 (2002) 617–637. doi: 10.1016/S0167-6105(02)00153-8

[32] Webb B.W., Hill D.P. (1989). High Rayleigh number laminar natural convection in an asymmetrically heated vertical channel. Journal of Heat Transfer 111:649–656.

[33] Mittelman G., Alshare A., Davidson J.H., (2009). Composite relation for laminar free convection in inclined channels with uniform heat flux boundaries, Int. J. Heat Mass Transfer 52, 4689–4694.

[34] Gan G., (2009), Numerical determination of adequate air gaps for building-integrated photovoltaics, Solar Energy, Vol 83, Issue 8, 2009, Pages 1253–1273. doi: 10.1016/j.solener.2009.02.008

[35] Dupont F., Ternat F., Samot S., Blonbou R., (2013), Two-dimension experimental study of the reverse flow in a free convection channel with active walls differentially heated, Experimental Thermal and Fluid Science, Vol 47,2013, 150–157. doi: 10.1016/j.expthermflusci.2013.01.010

